# Underwater Target Recognition Method Based on Singular Spectrum Analysis and Channel Attention Convolutional Neural Network

**DOI:** 10.3390/s25082573

**Published:** 2025-04-18

**Authors:** Fang Ji, Shaoqing Lu, Junshuai Ni, Ziming Li, Weijia Feng

**Affiliations:** China Ship Research and Development Academy, Beijing 100101, China

**Keywords:** underwater acoustic target recognition, singular spectrum analysis, channel attention mechanism, convolutional neural network

## Abstract

In order to improve the efficiency of the deep network model in processing the radiated noise signals of underwater acoustic targets, this paper introduces a Singular Spectrum Analysis and Channel Attention Convolutional Neural Network (SSA-CACNN) model. The front end of the model is designed as an SSA filter, and its input is the time-domain signal that has undergone simple preprocessing. The SSA method is utilized to separate the noise efficiently and reliably from useful signals. The first three orders of useful signals are then fed into the CACNN model, which has a convolutional layer set up at the beginning of the model to further remove noise from the signal. Then, the attention of the model to the feature signal channels is enhanced through the combination of multiple groups of convolutional operations and the channel attention mechanism, which facilitates the model’s ability to discern the essential characteristics of the underwater acoustic signals and improve the target recognition rate. Experimental Results: The signal reconstructed by the first three-order waveforms at the front end of the SSA-CACNN model proposed in this paper can retain most of the features of the target. In the experimental verification using the ShipsEar dataset, the model achieved a recognition accuracy of 98.64%. The model’s parameter count of 0.26 M was notably lower than that of other comparable deep models, indicating a more efficient use of resources. Additionally, the SSA-CACNN model had a certain degree of robustness to noise, with a correct recognition rate of 84.61% maintained when the signal-to-noise ratio (SNR) was −10 dB. Finally, the pre-trained SSA-CACNN model on the ShipsEar dataset was transferred to the DeepShip dataset with a recognition accuracy of 94.98%.

## 1. Introduction

The classification and recognition of underwater acoustic target features is a significant research area in the field of passive sonar. Its primary applications span various industries, including fish species classification, oil and gas exploration, and ship classification. By classifying and identifying underwater acoustic target features, substantial human and material resources can be saved, reducing costs and creating higher value. In recent years, deep learning methods have been actively introduced into various fields, including facial recognition, speech recognition, and artificial intelligence, achieving remarkable success. Compared to traditional methods, the advantages of the hydroacoustic target feature classification and recognition model combined with deep learning methods are obvious.

Currently, deep learning models used in the field of underwater acoustic target recognition include Convolutional Neural Networks (CNNs) [1,2,3], Recurrent Neural Networks (RNNs), Deep Belief Networks (DBNs), Generative Adversarial Networks (GANs), and Autoencoder Neural Network (ANNs) [4]. Overall, feature extraction and classification recognition of hydroacoustic targets based on deep learning can be divided into two aspects: one is to transfer the collected hydroacoustic signals to the frequency domain and use the two-dimensional spectrograms for feature extraction and recognition, and the other is to carry out feature extraction and recognition of one-dimensional acoustic signals, and the two methods have their own advantages. Applying deep neural networks to the recognition of two-dimensional sound spectrograms is similar to their application in the field of image recognition. Specifically, the powerful feature extraction and learning capabilities of deep models are leveraged to extract deep features, such as characteristic line spectra, from two-dimensional spectrogram images that reflect target signals. For one-dimensional acoustic signals, feature recognition can be performed directly on the raw or processed one-dimensional spectra. Compared to traditional feature extraction and recognition methods, these approaches exhibit significant advantages, including faster recognition speed, higher recognition efficiency, and enhanced processing capabilities for large-scale data signals after training, which have gained considerable popularity among researchers in recent years.

Empirical Mode Decomposition (EMD) [5], Ensemble Empirical Mode Decomposition (EEMD) [6], Variational Mode Decomposition (VMD) [7], Local Mean Decomposition (LMD) [8], and Singular Spectrum Analysis (SSA) [9] are some of the most frequently used signal decomposition methods. Each of these methods has its own strengths and weaknesses, and they are all actively utilized in the field of signal decomposition. Among them, the SSA method is distinguished by its high processing speed, good stability, and advantages in low-frequency applications [10], making it widely applicable across multiple domains.

Singular Spectrum Analysis (SSA) is a nonparametric method for processing nonlinear time series data. It does not require classical assumptions about the stationarity or normality of residuals. By performing operations such as Singular Value Decomposition (SVD), grouping, and reconstruction on the trajectory matrix of the original time series, SSA extracts different component sequences from the time series, including noise signals, low-frequency signals, long-term trends, and seasonal trends. Furthermore, SSA can use the reconstructed noise-removed series to predict new data points, achieving goals such as time series forecasting, denoising, dimensionality reduction, and signal decomposition without knowing the parametric model of the time series under consideration.

SSA has been extensively applied in various fields. Golyandina [11] investigated the application of SSA to time series with missing data, enabling the extraction of additional components such as trend and periodic components while simultaneously filling in the missing data. Jain [12] proposed the Multivariate Auto-SSA (MA-SSA) and Multivariate Sliding Window SSA (MSM-SSA) algorithms for decomposing multisensor time series and multichannel signals, respectively.

When signals contain time-varying components, MA-SSA can only recover stable components, such as sinusoidal curves, while nonstable components remain mixed. The MA-SSA algorithm is applied to segments of the signal determined by sliding windows and step sizes rather than the entire length of the signal. Haghbin [13] developed Functional SSA (FSSA) based on the ideas of Multivariate Functional Principal Component Analysis (MFPCA) and univariate SSA to analyze functional time series. This method serves as a three-dimensional dimensionality reduction tool for decomposing functional time series and also as a visualization tool to illustrate concepts of seasonality and periodicity in functional spaces over time. Guo [14] combined methods such as EEMD, NLMs, WT, and SSA to achieve the denoising of target signals, with SSA primarily focusing on low-frequency signal characteristics. Gu [15] introduced a windowing technique in SSA, replacing the default rectangular window with an adjustable tapered window to produce reconstructed components with more concentrated energy, called Generalized Singular Spectrum Analysis (GSSA), used for the decomposition and analysis of nonstationary signals. Lei [16] and Dai [17] utilized singular values in processing underwater acoustic targets, demonstrating to some extent the potential of SSA in the field of underwater acoustic target recognition.

In the field of underwater acoustic target recognition, many scholars have actively explored and proposed a variety of deep network models. Chen [18] combined ResNet architecture with a channel attention module, which enhanced the useful features and suppressed the noise interference through the processing of the feature map channel information, and achieved a high recognition accuracy in the specific SNR interval of the ShipsEar dataset. HUAT [19], CFTANet [20], ARescat [21], CTA-RDnet [22], and other models were based on the idea of combining the attention mechanism with a specific network. Among them, the HUAT model, by leveraging cyclic smooth analysis, a multihead self-attention module, a CNN module, and Swin Transformer had a classification accuracy of 98.62% on the ShipsEar dataset; the CFTANet model, with the use of subband-connected Mel spectrograms and the multidomain attention mechanism, also obtained a favorable recognition result of 96.4% on this dataset. The ARescat model combined the residual connectivity network with the SE attention mechanism and used Focal Loss to address the dataset imbalance problem, achieving 95.8% recognition accuracy on the ShipsEar database; the CTA-RDnet model fused ResNet and DenseNet, incorporated channel and time attention mechanisms, used the original time-domain data as input, and demonstrated excellent performance in experiments under different working conditions. The 1DCTN model proposed by Yang [23] combined the advantages of 1D CNNs and Transformer and directly used the original time-domain signals as input, achieving a recognition accuracy of 96.84% on the ShipsEar dataset. The MR-CNN-A model proposed by Ma [24] tackled the data imbalance problem by using a multiscale convolutional kernel, attention mechanism and CFWCEL, which was stable and well adapted under different noise levels and data imbalance degrees. Feng [25] proposed a UATR-transformer model featuring a convolution-free Transformer architecture, which inputs acoustic waveforms after transforming them into log Mel-fbank features, perceived global and local information using the Multihead Self Attention (MHSA) mechanism, and used a T-F Tokens Pooling classifier instead of the traditional classification approach. An average overall accuracy of 96.9% and 95.3% was achieved on the ShipsEar and Deepship datasets, respectively.

SSA has been widely applied in various fields over the past two decades, including weather forecasting, power load forecasting, and financial risk prediction. However, its application in underwater acoustic signal processing has been relatively limited. This paper explores this direction and conducts recognition experiments using publicly available datasets to evaluate the performance of the proposed model.

The notable characteristics of underwater acoustic target signals are primarily concentrated in the lower frequency range. Therefore, introducing SSA into hydroacoustic target feature classification and recognition has inherent advantages.

This paper proposes a hydroacoustic target feature classification and recognition model that combines SSA with a Channel Attention Convolutional Neural Network (CACNN). The front end of this model uses the SSA method to decompose the original time-domain signal into multi-order components. This process enables the effective separation of useful signals from irrelevant noise. A relatively small number of reconstruction orders reduce the complexity of the network and increase the calculation speed of the network. The back end of the model incorporates a channel attention mechanism, which improves the model’s ability to extract essential features of the target, thereby enhancing recognition performance and increasing the model’s robustness.

The structure of the paper is as follows:

Part 1: The introduction provides a comprehensive overview of the research background and objectives. Part 2 is a theoretical introduction to the principles of SSA, CNN, and Channel Attention (CA). Part 3 is a detailed description of the proposed SSA-CACNN model. Part 4 presents the analysis of the recognition results of the proposed model on real-world datasets. Part 5: The conclusion summarizes the findings and contributions of the study.

The main contributions of the paper are as follows:

(1) The paper proposes a novel approach that integrates the conventional rapid SSA signal decomposition method with the deep attention convolutional neural network. This integration contributes to enhancing the efficiency of underwater acoustic target recognition. The model exhibits a certain degree of robustness in the case of a relatively low SNR.
(2) The front end of the SSA-CACNN deep neural network model uses the SSA method that can directly process raw time-domain underwater acoustic signals. The decomposition process significantly reduces the impact of noise. The back end incorporates a channel attention mechanism to weight signal features, enhancing the model’s ability to extract the essential characteristics of the signals.
(3) In the SSA-CACNN deep neural network model, the front-end SSA component requires only the first three components to reconstruct the original signal. Compared to other methods, this approach helps reduce the number of network parameters and lowers the overall complexity of the network.

## 2. SSA Module Design and Principles

### 2.1. Signal Preprocessing Module

The time-domain noise signals of underwater acoustic targets, such as cruise ships and cargo ships, are collected by a passive signal acquisition system:

(1) $$x(n)=[x(1),x(2),\ldots ,x(f_{x}),\ldots ,x(M)]$$

where $f_{x}$ is the signal sampling rate and M is the number of time-domain sampling points of the signal.

The time-domain radiated noise signals of underwater acoustic targets, such as cruise ships and cargo ships, collected by the passive signal acquisition system are resampled to obtain time-domain signals with the same sampling rate:

(2) $$x(k)=resample(x(n),f_{x},f_{t})$$

where x(k) is the resampled standard signal, resample is the resampling algorithm in the corresponding programming software, which could be quadratic spline interpolation or others, $f_{s}$ is the sampling rate of the original signal, and $f_{t}$ is the sampling rate of the resampled signal.

After resampling, the input noise signals become more regular, which facilitate the input to the subsequent network models for further processing.

First, the resampled time-domain signals are preprocessed through framing, resulting in the following framed signals:

(3) $$X^{K\times L}=\left[\begin{array}{c} X_{0}\\ X_{1}\\ \vdots \\ X_{K-1} \end{array}\right]=\left[\begin{array}{cccc} x(0\times P+1)\; & x(0\times P+2)\; & \cdots & x(0\times P+L)\;\\ x(1\times P+1)\; & x(1\times P+2) & \cdots & x(1\times P+L)\\ \vdots & \vdots & \ddots & \vdots \\ x((K-1)\times P+1) & x((K-1)\times P+2) & \cdots & x((K-1)\times P+L) \end{array}\right]$$

where L is the number of sampling points per frame, and *K* is the number of frames. Specific parameters can be set according to actual requirements. In the experiment, each frame is set to consist of 4096 sampling points, with a 50% overlap between adjacent frames. This effectively eliminates the edge effects and facilitates subsequent signal reconstruction.

Then, the resampled sub-frame signals are zero-averaged [26]:

(4) x(p)=x(i)−μ

(5) $$\mu =\frac{1}{4096}\sum_{i=1}^{4096}x(i)\;\;\;\;i=1,2,\ldots ,4096$$

where x(i) is the value at each sampling point of a frame of the signal after resampling, x(p) is the value at each sampling point of a frame of the signal after zero-averaging, and μ is the mean value of a frame of the signal.

Finally, the signal is normalized to obtain the split-frame signal for subsequent filtering as follows:

(6) $$x^{\prime }(p)=\frac{x(p)}{\max(|x(p)|)}\;\;\;\;p=1,2,\ldots ,4096$$

where $x^{\prime }(p)$ is a normalized frame of signal that is used as input to the subsequent model.

### 2.2. Decomposition and Reconstruction Module

The front end of the SSA-CACNN model utilizes SSA to decompose and reconstruct one-dimensional time-domain signals. Processing the signal through this method can eliminate the influence of high-frequency noise, resulting in smoother signals while retaining significant low-frequency features.

The signal after resampling and framing is processed by singular spectrum analysis to obtain the radiated noise signal of the hydroacoustic target after denoising, which mainly contains four steps: embedding, decomposition, grouping, and reconstruction [27].

As shown in Table 1, the first step is to perform an embedding operation on the time series signal, which results in a trajectory matrix. Specifically, assuming the length of the time series is *N*, we have:

(7) $$X=\{x_{n}\}(n=1,2,\ldots ,N)$$

For ease of observation, it can be written as:

(8) $$x_{n}=[x_{1},x_{2},\ldots ,x_{N}]$$

Set up a window of length *M*, generally ordered as M<N/2. Use this window to intercept the signal and set each step to move a position so that each step will obtain a length of *M* time series as a column of the matrix. After the length of the *N* signal sliding interception, an M×(N−M+1) matrix will be formed, known as the ‘trajectory matrix’:

(9) $$Y=\left[\begin{array}{cccc} x_{1} & x_{2} & \cdots & x_{N-M+1}\\ x_{2} & x_{3} & \cdots & x_{N-M+2}\\ & & \vdots & \\ x_{L} & x_{L+1} & \cdots & x_{N} \end{array}\right]$$

Let K=N−M+1; we have the following:

(10) $$Y=\left[\begin{array}{cccc} x_{1} & x_{2} & \cdots & x_{K}\\ x_{2} & x_{3} & \cdots & x_{K+1}\\ & & \vdots & \\ x_{L} & x_{L+1} & \cdots & x_{N} \end{array}\right]$$

**Figure 1:**
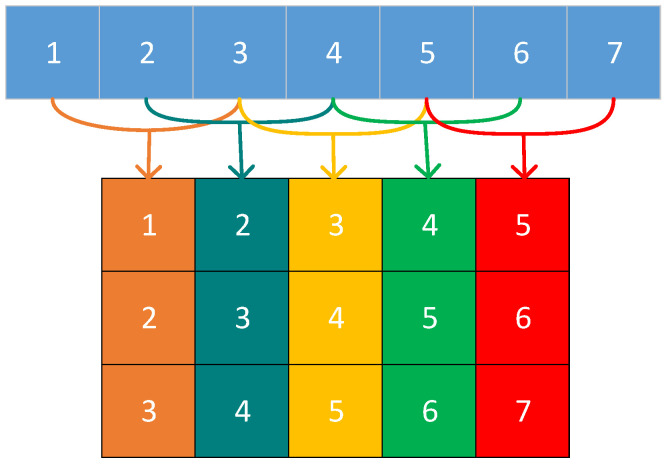
Example of windowed signal to raw signal decomposition.

Figure 1 shows an example of a trajectory matrix obtained from embedding a signal with a sequence length of 7, and a window length of 3 is given to illustrate this step more graphically.

Next, Singular Value Decomposition (SVD) is performed on the trajectory matrix obtained in the previous step.

Then, divide the singular values into m groups, denoted as:

(11) $$\left\{\alpha_{1},\alpha_{2},\ldots ,\alpha_{m}\right\}$$

The purpose is to decompose the matrix Y into the sum of linearly independent submatrices, i.e.,

(12) $$Y=Y_{1}+Y_{2}+\ldots +Y_{m}$$

Finally, reconstruct the signal. This step can be achieved by calculating the projection of $Y_{i}$ onto each group $U_{m}$:

(13) $$t_{i}^{m}=Y_{i}U_{m}=\sum_{j=1}^{L}x_{i+j}U_{m,j}\;\;\;\;0\leq i\leq N-L$$

where $Y_{i}$ represents the i-th column of the trajectory matrix Y, and $t_{i}^{m}$ reflects the weights of the time evolution type reflected by $Y_{i}$ in a certain period of the original sequence $x_{i+1}$, $x_{i+2}$, …, $x_{i+L}$, which are the temporal principal components. The signal is then reconstructed using the temporal empirical orthogonal functions and temporal principal components. By summing up all the reconstructed sequences, the original sequence before embedding can be obtained:

(14) $$x_{i}=\sum_{k=1}^{L}x_{i}^{k}\;\;\;\;i=1,2,\ldots ,N$$

However, in the application of this paper, only the first few orders of signal waveforms are needed, while the subsequent noise components are unnecessary.

**Figure 2:**
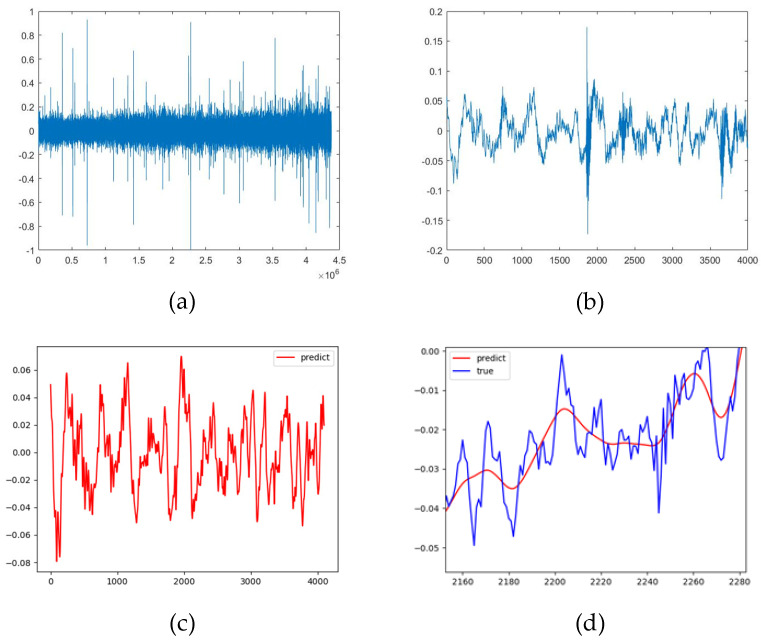
Signal decomposition and reconstruction of waveforms, (**a**) a segment of the time-domain signal of a surface vessel target collected by the passive signal acquisition system; (**b**) the first frame of the preprocessed time-domain signal; (**c**) the first frame of the signal after denoising using the SSA algorithm; (**d**) the local comparison.

**Figure 3:**
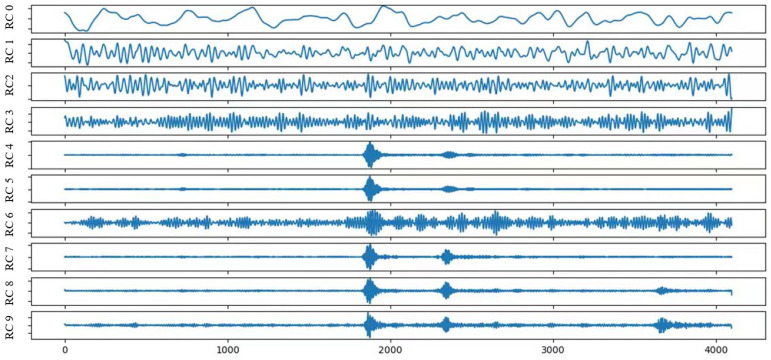
Waveform of the first nine orders of the signal after SSA decomposition.

Figure 2 and Figure 3 show the signal waveforms before and after SSA decomposition, in which Figure 2a shows a segment of the time-domain signal of the surface vessel target obtained by the passive signal acquisition system; Figure 2b shows the first frame of the time-domain signal preprocessed by Figure 2a; Figure 2c is the signal after denoising by SSA algorithm in Figure 2b, and it can be clearly observed that the high-intensity component in the middle part has been removed and the overall signal is smoother than before; Figure 2d shows a local comparison figure, which shows the details of the 128 sampling points of Figure 2b,c at the same time, blue is the original signal, and red is the signal processed by the SSA algorithm. The changes in the signal before and after the decomposition and reconstruction of the SSA algorithm can be seen more clearly, with the high-frequency fluctuations being smoothed out, highlighting the low-frequency features of the signal. Figure 3 shows the waveforms of the first 10th-order signal waveforms after embedding, decomposition, and grouping.

## 3. CACNN Model Construction

### 3.1. Fundamental Principles of Convolutional Neural Network (CNN)

CNN is typically composed of convolutional layers, pooling layers, activation functions, fully connected layers, and classification layers.

1. Convolutional layer

The convolutional layer can be categorized into one-dimensional (1D) and two-dimensional (2D) convolutional layers. For time series data, 1D convolution has a strong inherent advantage. The input of 1D convolution consists of a vector and a convolutional kernel, and the output is also a vector. Typically, the length of the input vector is much larger than the length of the convolutional kernel. The length of the output vector depends on the padding scheme used in the convolution operation. In the case of equal-width convolution, the length of the output vector is equal to that of the input vector. The length of the convolutional kernel, which is designed for symmetry, is usually odd.

2. Pooling Layer

The main function of the pooling layer is to reduce the feature dimension and the complexity of the neural network, but it also has the effect of improving the generalization ability of the model and alleviating the overfitting problem. The attention mechanism used in this paper is mainly realized by adjusting the scope of the pooling layer. Pooling can be divided into general pooling and global pooling. The effect of global pooling is stronger, which can directly compress the features of different feature channels and generate the weight coefficients of the corresponding channels. Common general pooling methods include Global Average Pooling (GAP), Global Max Pooling (GMP), Global Energy Pooling (GEP) [28], etc.

3. Activation Function

**Figure 4:**
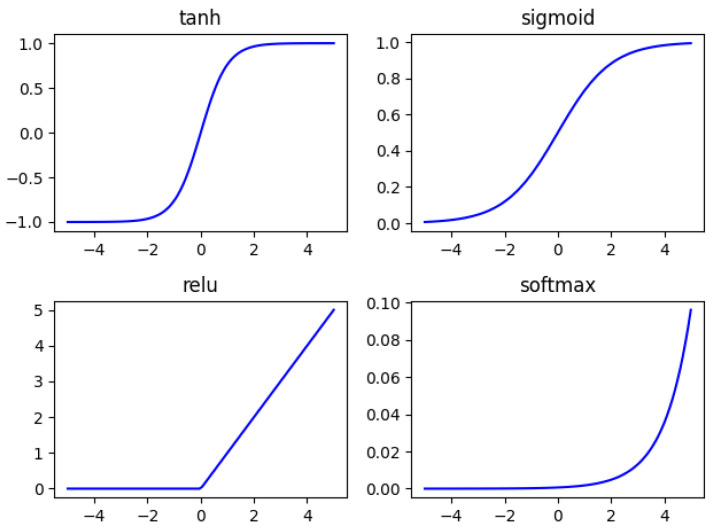
Activation function.

**Figure 5:**
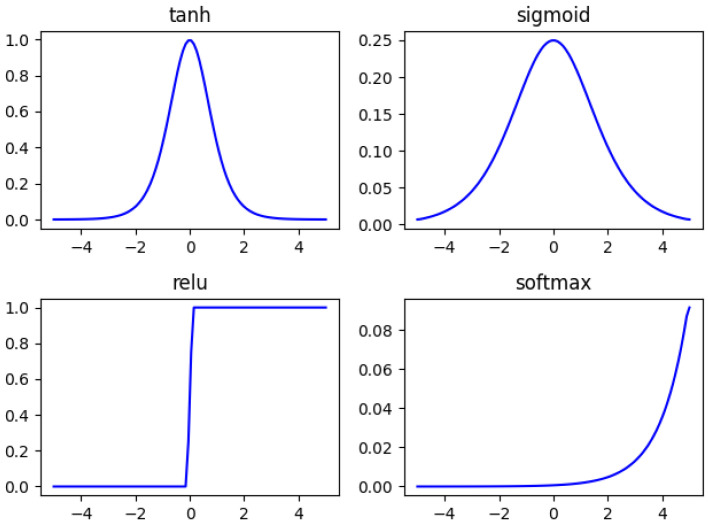
The derivative of the activation function.

The activation function plays an extremely important role in neural networks. In early intelligent methods, the sigmoid activation function is used in the BP neural network, which can limit the data between 0 and 1 while providing nonlinearity. However, because the derivative of this function takes a very small range of values, when the number of layers in the neural network increases, under the superimposed effect of multiple sigmoid functions, the problem of gradient vanishing is likely to occur, and the parameters of the BP neural network cannot be optimized. Therefore, in deep neural networks with a large number of layers, multiple sigmoid functions are usually not used consecutively. Instead, they are applied in combination with functions such as the tanh function and the ReLU function. Figure 4 shows several commonly used activation functions, and Figure 5 shows the derivatives of several commonly used activation functions.

(15) $$\left\{\begin{array}{ll} \mathrm{sigmoid}:\; & \frac{1}{e^{x}+1}\\ \tan h:\; & \frac{e^{x}-e^{-x}}{e^{x}+e^{-x}}\\ \mathrm{relu}: & \max(0,x)\;\\ \mathrm{softmax}:\; & \frac{e^{x_{i}}}{sum(e^{x_{i}})}\;,\;i=1,2,\ldots ,N \end{array}\right.$$

where x is the input signal and i is the location of the element.

4. Fully Connected Layer

The fully connected layer is generally used for the final classification task, the function of which is to take the output of the previous layer as input for combining all the local features extracted previously. Usually, the number of classification categories is set as the number of output nodes. Right after that, an activation function is applied to introduce nonlinearity so as to prevent the neural network model from overfitting.

5. Classification Layer

After the fully connected layer, all features form an output according to the number of classification categories. This output then goes through the softmax classification layer. According to Equation (13), after the vector data pass through the softmax function, it will result in a number between 0 and 1, which is the probability that the target is of each kind, and the neural network model determines the kind of the target by the size of the probability.

### 3.2. Attention Mechanism Module

Channel Attention (CA) [29] is one of the commonly used attention mechanisms at present. The main function of CA is to create weights for the features of different input channels. Usually, after the data have been convolved with a convolutional layer, the features of each channel have the same ‘importance’ in the following. Such a structure can extract the features in the signal and has a denoising effect, but the effect is limited. After adding CA, the model will assign different weights to different channels, making its “attention” more concentrated on the useful channels and reflecting the essential features of the target, enabling these “important” channels to play a greater role while reducing the effect of the “unimportant” channels.

**Figure 6:**
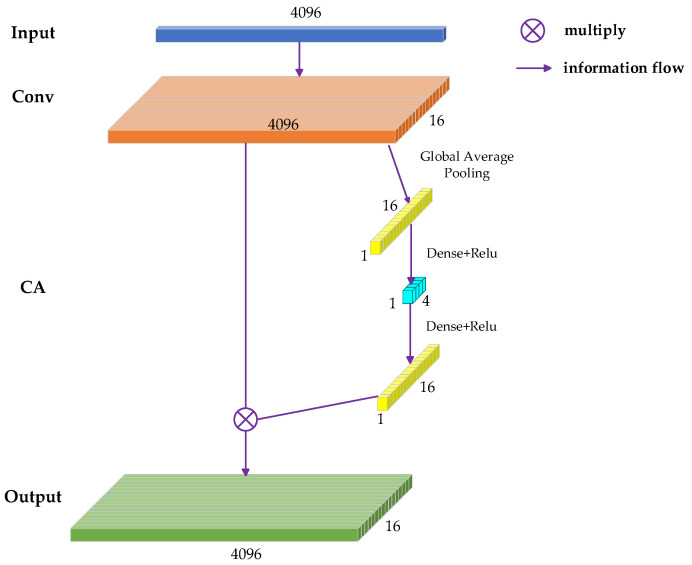
Channel Attention.

Figure 6 shows the model of CA channel construction. As can be seen from the figure, in the design of this module, the so-called “attention” of CA is mainly provided by the global pooling layer (Global Average Pooling, GAP; Global Max Pooling, GMP). The input data undergo 1D convolution, resulting in 16 feature channels, with each channel having 4096 dimensions. After global pooling, the 4096-dimensional data in each channel are compressed to one dimension, thereby generating a 16 × 1 feature weight, which is the core of CA, and this set of weights is recombined with the previous network layer before global pooling to get the weighted output. In this paper, the attention mechanism is added after the SSA decomposition, enabling the model to allocate more “attention” to the useful feature components to increase the weights of the feature channels, which play a specific positive role in the subsequent feature extraction and target recognition.

### 3.3. SSA-CACNN-Based Underwater Acoustic Target Recognition Model

**Figure 7:**
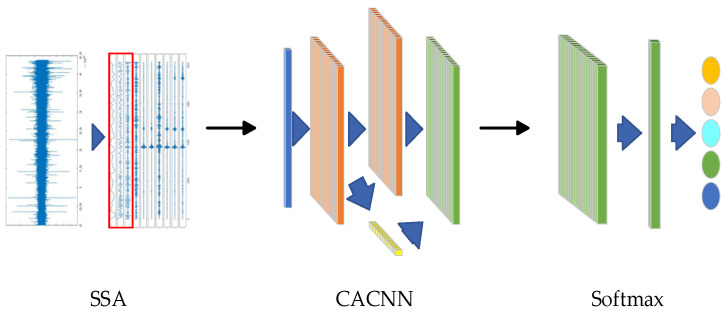
SSA-CACNN.

In reference [26], the research team of this paper proposed a method for underwater acoustic target recognition based on the Deep Residual Attention Convolutional Neural Network (DRACNN) in July 2023. This method achieved a recognition accuracy of 97.1% on the ShipsEar dataset. In this paper, the signal decomposition function of the SSA module is used to remove the high-frequency noise of the time-domain signal and improve the SNR of the signal. The CA mechanism of the DRACNN model is used to complete the abstract feature extraction and decision-making process. The subsequent convolutional model is composed of multiple Residual Attention Convolutional Blocks (RACB) connected in series. The dimension of its input data is the same as that of the output data of the SSA module. By connecting these modules in series, a new SSA-CACNN model is constructed. Finally, the underwater acoustic target recognition is achieved through a Softmax classifier. The structure of the SSA-CACNN model is shown in Figure 7, and the parameter data are presented in Table 2.

## 4. Experimental Results Analysis

### 4.1. Dataset and Sample Set

In this paper, the ShipsEar public dataset [30] was adopted as a validation dataset for target classification and recognition of the SSA-CACNN model. This dataset was sourced from http://atlanttic.uvigo.es/underwaternoise/ (accessed on 15 March 2023).

The recordings in the ShipsEar dataset were collected in the autumn of 2012 and the summer of 2013 at the port of VIGO and its vicinity on the northwest Atlantic coast of Spain. These recordings were obtained by using the autonomous acoustic digitalHyd SR-1 hydrophone manufactured by MarSensing Lda (Faro, Portugal), and recorded the noise of 11 types of ships, such as fishing vessels, ocean liners, ferries of various sizes, container ships, roll-on/roll-off ships, tugboats, pilot boats, yachts, and small sailing boats. Eventually, based on the sizes of the ships, they were merged into five categories, including four experimental ship categories and one background noise category, as shown in Table 3.

This dataset included 90 recordings in WAV format, with each recording duration ranging from 15 s to 10 min, for a total recording duration of 3.15 h, recorded at a sampling rate of 52,734 Hz.

In order to verify the model’s ability to classify and recognize the original time-domain signals, the original data of the dataset used in this paper was cut using the frame division method mentioned in Section 2.1 before being input into the model, which was designed to facilitate the unification of the data structure of the input model and to normalize the data, and no other enhancement processing was carried out on the time-domain signals. The dataset for the verification of this model was composed of the obtained 16,537 sample data, which were divided into 13,230 training data and 3307 test data according to the ratio of 8:2.

### 4.2. Experimental Results and Analysis

The SSA-CACNN model proposed in this paper was a deep-network model consisting of an SSA decomposition algorithm at the front end, an attention mechanism residual network in the middle, and a cognition network at the end, which was built in Python language and TensorFlow framework. The specific software environment was as follows: Windows 7 operating system, Python = 3.6.5, Keras GPU = 2.2.4, TensorFlow = 1.14.0, Cudatoolkit = 10.0.130, and Cudnn = 7.6.5. The hardware condition was a workstation equipped with an NVIDIA RTX2080Ti GPU, an Intel Xeon silver 4214R CPU, and 32 G of RAM.

**Figure 8:**
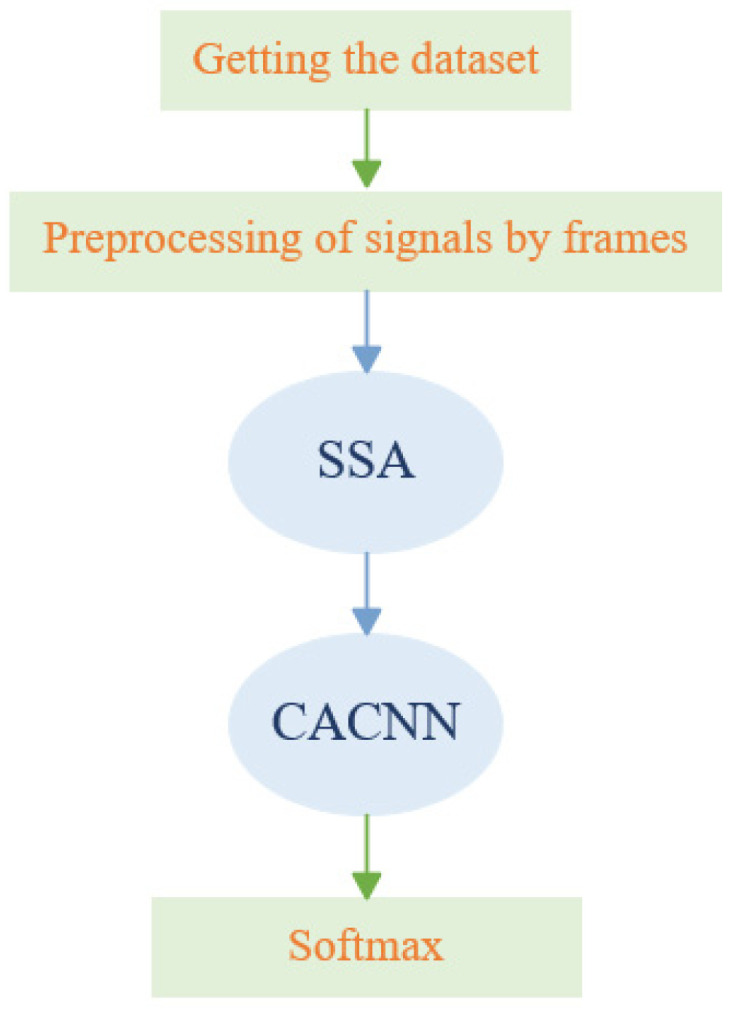
SSA-CACNN processing flow.

The built model was trained by setting the learning rate to 0.0005, the number of iterations to 100, the batch size to 64, the cost function to cross-entropy loss function, and the optimizer to Adam. The exponential decay rate of the first moment estimate was set to 0.9, and the exponential decay rate of the second moment estimate was set to 0.999. The SSA attention deep network model was trained using the training set samples, and the model parameters were optimized iteratively to drive the model to extract discriminative target features and establish a nonlinear mapping relationship between the input data and target types. The model was optimally trained based on the gradient descent algorithm, and the fitting performance of the model was verified by using the test set during the training process. The processing procedure of the method proposed in this paper is shown in Figure 8. First, an underwater acoustic target audio dataset with known labels was obtained. Second, the audio data were framed and integrated to construct a sample set that is convenient for the input of the network model. Third, the SSA algorithm at the front end of the model was used to decompose and reconstruct the samples to remove part of the noise. Then, the channel attention convolutional neural network was used to weight and extract the deep features. Finally, the softmax layer was used to discriminate the targets. In this paper, after 10 independent experiments using the complete ShipsEar dataset, the average classification and recognition result was 98.64%.

#### 4.2.1. The Classification and Recognition Results of the Model

**Figure 9:**
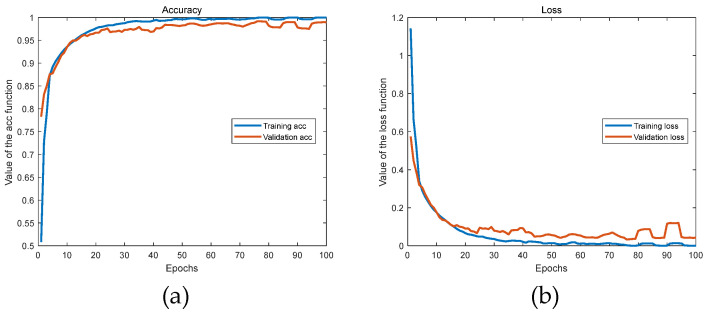
Accuracy curves and loss curves. (**a**) The training and validation recognition accuracies; (**b**) the training and validation transfer losses.

In this paper, the classification and recognition performance of the model was characterized by means of loss curves, training and testing curves, recognition results, confusion matrices, etc. The SSA part at the front end of the SSA-CACNN model decomposed the signal into 10 orders, and the first three orders of signals were taken as inputs to the model for reconstruction. The learning curve of the model was recorded, as shown in Figure 9. Figure 9a shows the training and validation recognition accuracies during the model learning process. The blue curve represented the change curve of the accuracy of the training set with the number of iterations, and the red curve represented the change curve of the accuracy of the validation set with the number of iterations. Figure 9b shows the training and validation transfer loss during the model learning process. The blue curve represented the training set’s transfer loss over iterations, and the red curve represented the validation set’s transfer loss over iterations. The final result of the model recognition in this paper was 98.64%.

**Figure 10:**
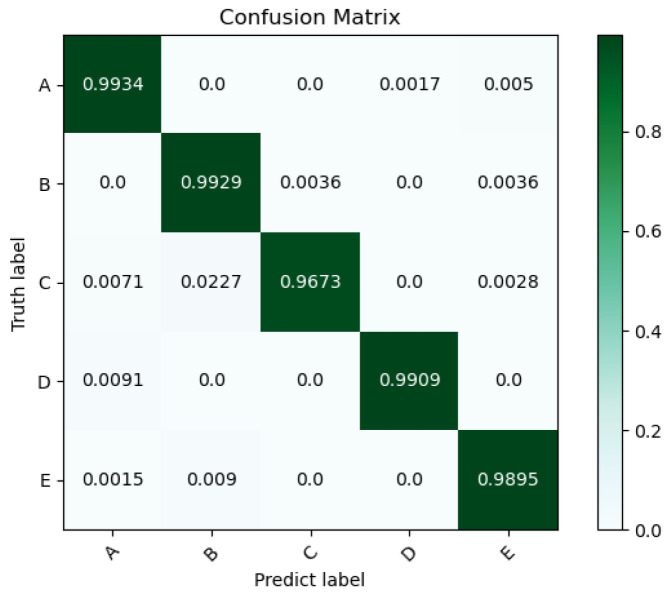
The confusion matrix of one of the recognition results.

In this paper, as shown in Figure 10, the experimental recognition accuracy was demonstrated using the confusion matrix, in which the dark green squares on the diagonal represented the correct recognition rate of the target classification, and the light blue squares on both sides were the misclassification of the target as other categories. It can be seen that among the five types of targets—A, B, C, D, and E—the recognition accuracies of targets A, B, and D were all above 99%, which was a considerable recognition result. The recognition accuracy of target E was slightly worse, and the recognition accuracy of target C was 96.73%, which was the worst among them. From the confusion matrix, it can be observed that most of the misclassifications of target C were recognized as target B, and a few were recognized as target A and target E, which may be because there was a specific overlap in the features between target C and these two types of targets. In addition, the uneven distribution of the number of targets in each category will also have an adverse effect on the recognition results.

In addition, this paper introduced several parameters commonly used in machine learning to characterize the recognition performance of the SSA-CACNN model more accurately and vividly. The calculation formulas for accuracy, precision, recall, and F1_score are shown as follows.

(16) $$Accuracy=\frac{TP+TN}{TP+TN+FP+FN}$$

(17) $$Precision=\frac{TP}{TP+FP}$$

(18) $$Recall=\frac{TP}{TP+FN}$$

(19) $$F1=2\times \frac{Precision\times Recall}{Precision+Recall}$$

where TP is true positive, TN is true negative, FP is false positive, and FN is false negative.

As shown in Table 4, this paper calculated the accuracy, precision, recall, F1_score and their average values for one of the recognition results. The F1_score can better illustrate the actual effect of a model compared to the accuracy. It can be seen that the values of target B and target C were relatively low, which was consistent with the feature aliasing of these two types of targets shown in the confusion matrix. Judging from the average scores of the five types of targets, the scores of targets B, C, and D were all lower than the average score. For the subsequent improvement of the model, research can be conducted on the similarities and differences of these types of targets, which is expected to improve the overall recognition rate of the model.

Table 5 shows the recognition accuracies and the number of parameters of the model proposed in this paper against several other models. All these models were verified for their performance using the ShipsEar dataset. The recognition accuracy of the SSA-CACNN model was 98.64%, only slightly lower than that of the ACNN_DRACNN model and the MR-CNN-A model. In addition, the Params of the SSA-CACNN model was 0.26 M, comparable to the Params of the DRACNN model among all the compared methods, and lower than all the other models for which the number of parameters can be known. To some extent, the differences in the model parameters can also reflect the different complexity of network models. Often, models of lower complexity are faster to compute and relatively more efficient under the same hardware and software conditions.

**Figure 11:**
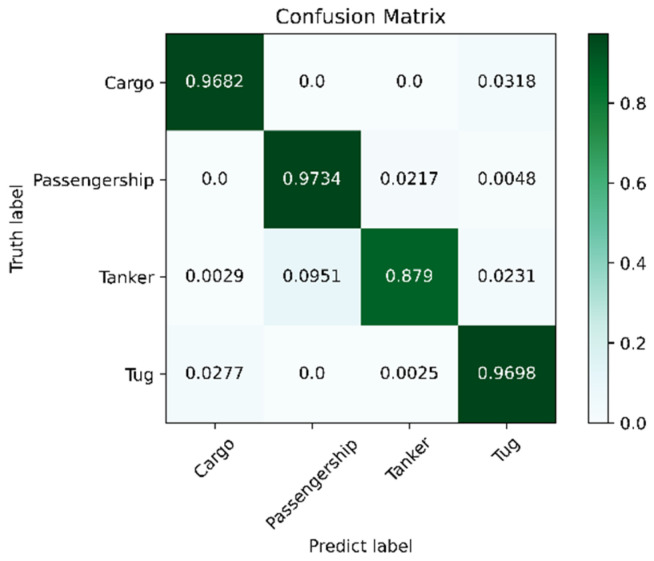
Confusion matrix plot for the transfer of the model to the DeepShip dataset.

The model trained with the ShipsEar dataset was transferred to the Deepship dataset to verify the generalization performance of the model. The DeepShip dataset [33] (https://github.com/irfankamboh/DeepShip/, retrieved on 1 July 2023) contains Cargo, Passenger Ship, Tanker, and Tug—four kinds of targets. In this experiment, three wav signals were extracted from each of the four categories. A total of 7676 samples of data were generated after preprocessing by the method shown in Section 2.1, and the training and test sets were divided according to the ratio of 8:2. The transfer method involved freezing most of the parameters of the model trained on the ShipsEar dataset and only unfreezing a few parameters such as the last four dense layers of the model, the Relu activation layer, and the Softmax classification layer, where the output dimension of the Softmax classification layer was 4. The SSA-CACNN model finally obtained a recognition accuracy of 94.98%. Figure 11 and Table 6 show the confusion matrix results of this transfer learning. Among the four targets, Tanker had the lowest recognition accuracy and F1-score. As shown in Figure 11, the Tanker samples were identified as Passenger Ship, which indicated that the vessels in the Tanker category have a high feature overlap with the Passenger Ship category. However, from the overall 94.98% recognition rate, SSA-CACNN had been able to achieve better generalization between the two datasets.

**Figure 12:**
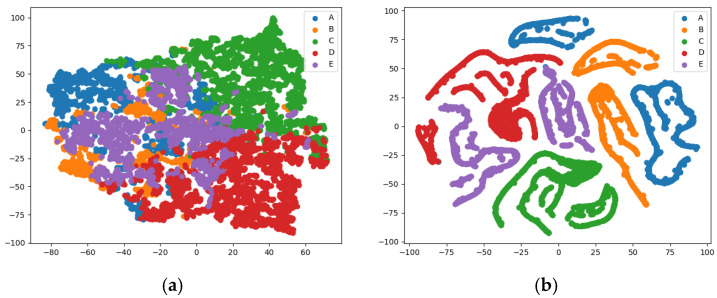

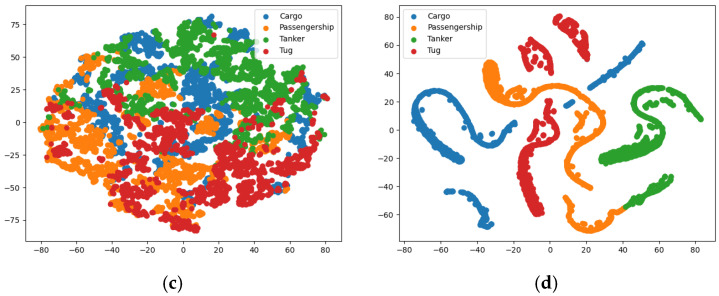
SSA-CACNN model 2D feature t-SNE visualization. (**a**) ShipsEar dataset feature visualization before model training; (**b**) ShipsEar dataset feature visualization after model training; (**c**) DeepShip dataset feature visualization before model transfer; (**d**) DeepShip dataset feature visualization after model transfer.

Figure 12a,b show the 2D t-SNE visualization results of the ShipsEar dataset sample output features before and after the training of the SSA-CACNN model, respectively. Before the SSA-CACNN training, the sample features were cluttered, and it was almost impossible to distinguish the five types of signals, while after the training, they were well differentiated, with only very few sample features overlapping. Subplots (c) and (d) of Figure 12 showed the 2D t-SNE visualization results of the sample output features of the SSA-CACNN model before and after transferring to the DeepShip dataset, respectively. Similar to the training result, the two categories of Tanker and Passenger Ship partially overlapped, and in general, the output features could be distinguished, which indicated that the SSA-CACNN model had some generalization performance.

#### 4.2.2. The Influence of the Order of Reconstructed Signals

The SSA module of the model proposed in this paper decomposes the signal, which forms multi-order components. In the process of reconstruction, if all signal components are superimposed, the complete original signal containing noise will be obtained, which is meaningless. Therefore, the order of the signal used in the actual reconstruction needs to be less than the order of the signal decomposed by the SSA. This paper studied the influence of the order of the SSA reconstructed signal on the deep network model.

In this paper, 5000 samples were selected from the ShipsEar as the dataset to study the influence of the order of signal reconstruction by the front-end SSA part on the neural network model. Among them, 4000 samples were used as the training set and 1000 samples were used as the test set. The samples of the five categories A, B, C, D, and E were selected with the same number of 1000 each in order to reduce the detrimental effect of sample inhomogeneity on the recognition results. Eighty percent of the samples in each category were used as the training set, and 20% were used as the test set. After 100 rounds of training and testing each time, the recognition accuracy of the signal decomposition model at this order is finally obtained. It can be seen that the model recognition accuracies obtained in this experiment were generally higher than the previous tests for verifying the model performance. This is because the number of samples selected in this experiment was extremely uniform and relatively small, resulting in a lower feature overlap rate between different categories. This was conducive to the model extracting and distinguishing the essential features between samples from different categories, so a higher accuracy rate was obtained. However, it should be noted that such an accuracy rate has no practical significance. The purpose of this experiment was only to verify the influence of the order of reconstructed signals on the deep network model to explore the advantages of the model better.

**Figure 13:**
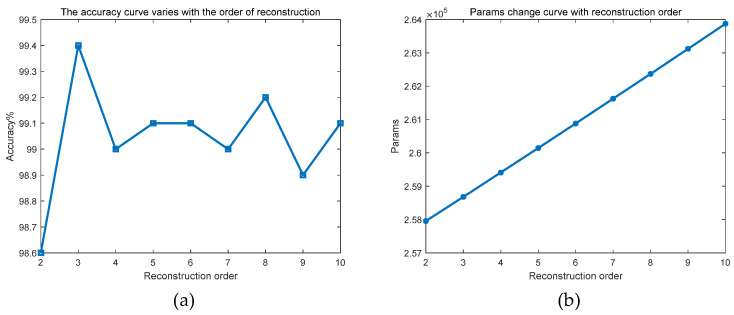
The effect of reconstructed signal order on the model. (**a**) The accuracy curve varies with the order of reconstruction; (**b**) the variation curve of Params with the order of reconstruction.

Table 7 and Figure 13 show the influence of the reconstruction order of the signal by the SSA part at the front end of the SSA-CACNN model. Figure 13a shows the accuracy curve varies with the order of reconstruction. As the reconstruction order of the signal increased, the recognition accuracy of the model did not improve. Instead, it reached the maximum value when the reconstruction order of the signal was 3. After that, as the reconstruction order of the signal increased, the accuracy fluctuated and decreased overall. This is because the SSA decomposed the features of the signal into the first three orders of components, and the signals after the third order contained relatively few actual features. Moreover, as the reconstruction order of the signal increased, the previously separated noise components were reconstructed into the new signal, so the recognition rate of the model decreased instead. Figure 13b shows the variation curve of Params with the order of reconstruction. Judging from the Params, as the reconstruction order of the signal increased, the Params of the model increased linearly, which undoubtedly increased the complexity of the network and had an adverse effect on the calculation speed of the model. Therefore, in a comprehensive view, when the reconstruction order of the signal of this model was 3, it can better separate the noise components, extract the target features, and maintain high accuracy. On the other hand, the model had a lower number of Params, which was conducive to improving the calculation speed and efficiency of the model when processing large datasets, helping to reduce the training cost to a certain extent.

#### 4.2.3. Robustness After Adding Noise

**Figure 14:**
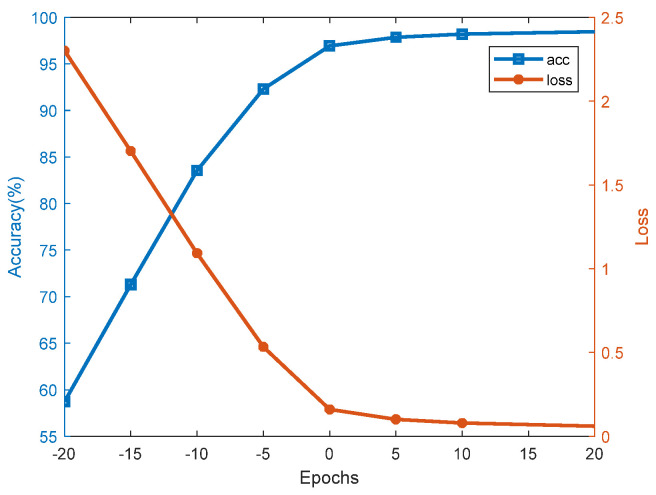
Effect of SNR on model recognition performance.

In order to explore the robustness of the SSA-CACNN model, Gaussian white noise with different SNRs was added to the sound signals of the original ShipsEar dataset to investigate the variation of the model recognition accuracy. Figure 14 shows the effect of SNR on the model recognition performance. The recognition rate decreased as the signal noise increased. The model maintained a recognition accuracy of 93.23% under the condition of an SNR of −5 dB and still 84.61% accuracy under −10 dB SNR. It can be seen that the model had a particular degree of robustness for target recognition after the addition of noise. However, when the SNR was lower than −15 dB, the recognition rate of the model dropped below 75%, and it was difficult to extract the features of the target.

## 5. Conclusions

This paper introduces the SSA-CACNN model based on the Singular Spectrum Analysis (SSA) method and the Deep Residual Attention Convolutional Neural Network (DRACNN). The input of the model is the original time-domain hydroacoustic signal. The SSA method focuses on extracting low-frequency signal features. After the decomposition and reconstruction, the impact of high-frequency noise on the signal is substantially reduced. Moreover, the SSA method only requires a small number of order components to reconstruct the original signal, significantly reducing the parameter count of the model and the complexity of the network. In experimental validation, the model achieved an average recognition rate of 98.64%. Additionally, it maintained a correct recognition rate of 84.61% under an SNR of −10 dB, demonstrating its robustness against noise. Finally, the trained SSA-CACNN model was transferred to the DeepShip dataset to evaluate its generalization performance, achieving a recognition accuracy of 94.98%.

However, the SSA method may remove some high-frequency signal features while eliminating high-frequency noise, which may have a negative effect on the classification and recognition of targets. Therefore, in future research, this paper intends to explore another method to extract high-frequency signal features. Fusing both low-frequency and high-frequency features for underwater acoustic target classification and recognition is expected to improve the model recognition rate, although this may increase the complexity of the model structure, which will be another research issue. In summary, when seeking a feature extraction classification model for hydroacoustic targets with a high recognition rate and good generalization, computational efficiency must also be considered—perhaps a balance between the two is a better approach.

## Figures and Tables

**Table 1:** Design of decomposition and reconfiguration module.

SSA Operating Procedures	
Step1	Embedding—forming a trajectory matrix.
Step2	Decomposition—SVD for signal decomposition.
Step3	Grouping—grouping the features.
Step4	Refactoring—obtain denoised signal after reconstruction (if all components are summed, the reconstructed signal will be the original signal, albeit with minor errors).

**Table 2:** Model structure.

Layer	Input Shape	Output Shape
Input	(None, 4096, 3)	(None, 4096, 3)
CA	(None, 4096, 3)	(None, 4096, 3) (None, 1, 3)
Conv1D	(None, 4096, 1)	(None, 4096, 16)
CA	(None, 4096, 16)	(None, 4096, 16) (None, 1, 16)
Conv1D	(None, 1024, 16)	(None, 1024, 32)
GAP	(None, 1024, 32)	(None, 32)
Conv1D	(None, 256, 32)	(None, 64, 128)
Conv1D	(None, 64, 128)	(None, 32, 128)
Conv1D	(None, 32, 128)	(None, 16, 128)
GAP	(None, 16, 128)	(None, 128)
Dense	(None, 128)	(None, 5)
Output	(None, 5)	(None, 5)

**Table 3:** ShipsEar dataset.

Category	Type of Vessel	Files	Duration (s)
Class A	Fishing boats, trawlers, mussel boats, tugboats, dredgers	17	1880
Class B	Motorboats, pilot boats, sailboats	19	1567
Class C	Passenger ferries	30	4276
Class D	Ocean liners, RORO vessels	12	2460
Class E	Background noise recordings	12	1145

**Table 4:** Detailed results of the SSA-CACNN model.

Categories	Accuracy (%)	Precision (%)	Recall (%)	F1-Score (%)
A	99.34	97.88	99.34	98.6
B	99.29	96.21	99.29	97.72
C	96.73	99.71	96.73	98.2
D	99.09	99.87	99.09	99.48
E	98.95	98.95	98.95	98.95
Average	98.68	98.52	98.68	98.59

**Table 5:** Test results based on the ShipsEar dataset.

No.	Method	Accuracy (%)	Params (M)
1	ResNet-18 [31]	94.9	0.78
2	AResNet [32]	98.0	9.47
3	DRACNN [26]	97.1	0.26
4	SE_ResNet [18]	98.09	\
5	HUAT [19]	98.62	30.3
6	CFTANet [20]	96.4	0.47
7	ARescat [21]	95.8	\
8	1DCTN [23]	96.84	0.45
9	MR-CNN-A [24]	98.87	\
10	UATR-transformer [25]	96.9	2.55
11	ACNN_DRACNN [28]	99.87	0.61
12	SSA-CACNN (the proposed method)	98.64	0.26

**Table 6:** Confusion matrix table for the transfer of the model to the DeepShip dataset.

Categories	Accuracy (%)	Precision (%)	Recall (%)	F1-Score (%)
Cargo	96.82	96.82	96.82	96.82
Passenger Ship	97.34	92.43	97.34	94.82
Tanker	87.9	96.83	87.9	92.15
Tug	96.98	94.59	96.98	95.77
Average	94.76	95.17	94.76	94.89

**Table 7:** The effect of different orders of reconstructed signals on the model.

Reconstruction Order	Accuracy	Params
2	98.6%	257,954
3	99.4%	258,680
4	99.0%	259,410
5	99.1%	260,144
6	99.1%	260,882
7	99.0%	261,624
8	99.2%	262,370
9	98.9%	263,120
10	99.1%	263,874

## Data Availability

Data are available in a publicly accessible repository. Datasets are openly available at http://atlanttic.uvigo.es/underwaternoise/ (ShipsEar, accessed on 15 March 2023) and https://github.com/irfankamboh/DeepShip/ (DeepShip, accessed on 1 July 2023).
